# Medial temporal pathways for contextual learning: Network c-*fos* mapping in rats with or without perirhinal cortex lesions

**DOI:** 10.1177/2398212817694167

**Published:** 2017-03-14

**Authors:** Lisa Kinnavane, Eman Amin, Cristian M. Olarte-Sánchez, John P. Aggleton

**Affiliations:** 1 School of Psychology, Cardiff University, Cardiff, UK; 2 The Institute of Medical Sciences, University of Aberdeen, Aberdeen, UK

**Keywords:** Entorhinal cortex, hippocampus, nucleus reuniens, prefrontal cortex, retrosplenial cortex, spatial memory, subiculum

## Abstract

**Background:**

In the rat brain, context information is thought to engage network interactions between the postrhinal cortex, medial entorhinal cortex, and the hippocampus. In contrast, object information is thought to be more reliant on perirhinal cortex and lateral entorhinal cortex interactions with the hippocampus.

**Method:**

The ‘context network’ was explored by mapping expression of the immediate-early gene, c-*fos*, after exposure to a new spatial environment.

**Results:**

Structural equation modelling of Fos counts produced networks of good fit that closely matched prior predictions based on anatomically grounded functional models. These same models did not, however, fit the Fos data from home-cage controls nor did they fit the corresponding data from a previous study exploring object recognition. These additional analyses highlight the specificity of the context network. The home-cage controls, meanwhile, showed raised levels of inter-area Fos correlations between the many sites examined, that is, their changes in Fos levels lacked anatomical specificity. A total of two additional groups of rats received perirhinal cortex lesions. While the loss of perirhinal cortex reduced lateral entorhinal c-*fos* expression, it did not affect mean levels of hippocampal c-*fos* expression. Similarly, overall c-*fos* expression in the prelimbic cortex, retrosplenial cortex, and nucleus reuniens of the thalamus appeared unaffected by the perirhinal cortex lesions.

**Conclusion:**

The perirhinal cortex lesions disrupted network interactions involving the medial entorhinal cortex and the hippocampus, highlighting ways in which perirhinal cortex might affect specific aspects of context learning.

## Introduction

**Figure 1.**
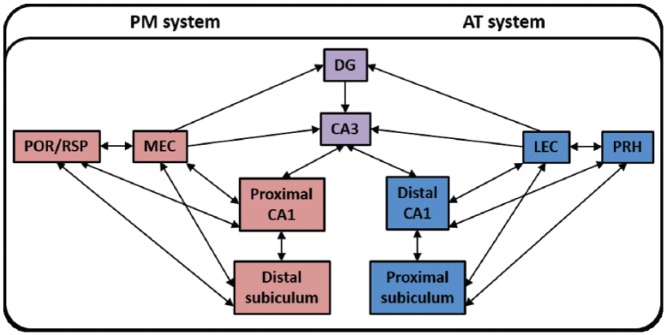
Posterior–medial (PM) context system and anterior–temporal (AT) item system of the binding of item and context (BIC) framework. Parallel cortico-hippocampal pathways link the PM and AT systems with the entorhinal cortex, CA1, and subiculum. Source: Adapted from Ritchey et al. (2015).

Models of medial temporal lobe processing increasingly assume two distinct functional pathways (Figure 1), one for object-based information and the other for spatial and contextual information (Bucci and Robinson, 2014; Burwell, 2000; Eacott and Gaffan, 2005; Diana et al., 2007; Knierim et al., 2014; Ranganath and Ritchey, 2012; Ritchey et al., 2015). In the case of the rodent brain, perirhinal cortex is presumed to process object-based information, in concert with the lateral entorhinal cortex (LEC; Barker et al., 2007; Mumby and Pinel, 1994; Naber et al., 1997). In contrast, postrhinal cortex, along with the medial entorhinal cortex (MEC), is presumed to provide spatial and contextual information for the hippocampus (Bucci et al., 2000; Bucci and Robinson, 2014; Burwell, 2000; Furtak et al., 2007; Norman and Eacott, 2005; Ranganath and Ritchey, 2012). These distinct, functional pathways are highlighted in a number of models, including the binding of item and context (BIC; Diana et al., 2007) framework as well as in subsequent anatomical refinements of this basic model (Aggleton, 2012; Ranganath and Ritchey, 2012; Ritchey et al., 2015).

This study quantified the expression of the immediate-early gene (IEG) c-*fos* after placing rats in a novel context in order to activate one of these processing pathways. This IEG, which provides an indirect marker of neural activity (Bisler et al., 2002; Chaudhuri, 1997; Zangenehpour and Chaudhuri, 2002), is known to show increased hippocampal activity following contextual change (e.g. Albrechet-Souza et al., 2011; Jenkins et al., 2004; Vann et al., 2000). The importance of this c-*fos* expression is strikingly highlighted by studies showing how reactivating those dorsal hippocampal neurons that had previously expressed c-*fos* in a distinctive context can reinstate representations of that same context (Liu et al., 2012, 2014; Ramirez et al., 2013). Structural equation modelling (SEM; McIntosh and Gonzalez-Lima, 1991; Schumacker and Lomax, 2010) was then used to test anatomically plausible models based on refinements of the BIC framework (Ritchey et al., 2015).

As already noted, current medial temporal models typically assume the presence of two parallel pathways that emanate, respectively, from the perirhinal and parahippocampal (postrhinal) cortices to reach the hippocampal formation (Diana et al., 2007; Witter, 2002). There are, however, reciprocal connections between the postrhinal and perirhinal cortices and between the MEC and LEC (Burwell and Amaral, 1998a, 1998b; Furtak et al., 2007). These interconnections question the degree of independence between these two functional pathways (Kent and Brown, 2012; Knierim et al., 2014; Liu and Bilkey, 1998b, 2001). For this reason, this study also examined how perirhinal cortex lesions might affect parahippocampal–hippocampal c-*fos* activity following exposure to a novel context. The question was whether the context pathway still shows normal activity patterns in the absence of perirhinal cortex. Finally, other intact rats and rats with perirhinal cortex lesions were examined for c-*fos* expression after receiving no context shift, so providing a comparison baseline condition.

## Materials and methods

### Animals

Subjects were 56 male, Lister Hooded rats (Harlan, Bicester, UK), housed in pairs under diurnal conditions (12-h light/12-h dark). The home cages measured 42 cm × 25 cm × 21 cm with a water bottle and food hopper at the front. Each cage, which had opaque plastic floors and walls (13 cm high), was lined with sawdust and contained a cardboard tube and chew stick. The rats, which were fed 2014 Teklad global 14% protein rodent maintenance diet (Harlan), were on an ad libitum schedule. All experiments were performed in accordance with the UK Animals (Scientific Procedures) Act, 1986, and associated guidelines and approved by local ethical committees at Cardiff University.

The rats came from two cohorts of animals, which received the same experimental protocols throughout the present experiment. Rats in cohort A (n = 29) were approximately 11 months old at the beginning of the c-*fos* imaging study. Of these rats, 18 had received perirhinal cortex lesions, while 11 served as their surgical controls. Rats from cohort B (n = 27) were approximately 7 months old at the beginning of the present experiment. Of these, 15 received perirhinal cortex lesions, while 12 received sham surgeries. Prior to the current experiment, both cohorts received object recognition memory tasks in the bow-tie maze (full details are described in Albasser et al., 2015). Additionally, both cohorts received a single, spontaneous object recognition test in an open-field apparatus. Rats were not behaviourally tested for at least 2 weeks before the current experiment, with water and food available ad libitum throughout this intervening period.

### Surgery

The rats were approximately 3 months old at the time of surgery, when they weighed between 285 and 300 g. In total, 33 rats received bilateral perirhinal cortex lesions (‘Peri’), while 23 rats served as surgical controls (‘Sham’). Anaesthesia was induced using a mixture of oxygen and isoflurane gas (5% for induction and 2% thereafter), before placing each rat in a stereotaxic frame (David Kopf Instruments, Tujunga, CA, USA), with the incisor bar set at +5.0 mm above the horizontal plane. After making a midline sagittal incision in the scalp, the skin was retracted to expose the skull. Following a craniotomy, the perirhinal lesions were made by injecting a solution of N-methyl-d-aspartate (NMDA; Sigma, Poole, UK) diluted to 0.09 M in phosphate-buffered saline (PBS; 0.1 M, pH 7.4) using a 1-µm Hamilton syringe (Bonaduz, Switzerland; gauge 26, outside diameter 0.47 mm) held with a micro-injector (model 5000; Kopf Instruments, Tujunga, CA). Bilateral injections of NMDA (each of 0.225 µL) were made at a rate of 0.10 µL/min, with a subsequent diffusion time of 4 min before the needle was removed. Three injections were made in each hemisphere. Injection coordinates relative to bregma (in mm) were (1) anterior–posterior (AP): −1.8, medial–lateral (ML): ±5.9, and dorsal–ventral (DV): −9.3; (2) AP: −3.4, ML: ±6.1, and DV: −9.6; (3) AP: −5.0, ML: ±6.2, and DV: −9.0. Rats in the surgical control group received identical treatments, except that the dura was perforated three times per hemisphere with a 25-gauge Microlance 3 needle (Becton Dickinson, Drogheda, Ireland) and no fluid was infused into the brain.

### Apparatus – activity boxes

For the novel context condition, rats were placed individually in an activity cage in a novel room (272 cm × 135 cm × 240cm). A 3 × 6 bank of activity cages was located along one wall of the room. Each activity cage (Paul Fray, Cambridge, UK) measured 56 cm × 39 cm × 19 cm and contained two photobeams placed 20 cm apart, positioned 18 cm from the short walls. The floor of each cage was made of wire; otherwise, the cage was empty. The top of each cage was also made of wire, and the room was illuminated.

### Behavioural testing

Both the Peri and Sham animals were divided between the two behavioural conditions, creating four groups. Rats with perirhinal lesions were assigned to either the novel context condition (Peri Novel, n = 18; nine from cohort A, nine from cohort B) or the home-cage control condition (Peri Baseline, n = 15; 9 from cohort A, 6 from cohort B). Similarly, the sham surgical controls were divided between the novel context condition (Sham Novel, n = 11; 4 from cohort A, 7 from cohort B) and the home-cage control condition (Sham Baseline, n = 12; 7 from cohort A, 5 from cohort B). Behavioural testing (activity boxes) took place either 8 (cohort A) or 4 (cohort B) months after surgery.

For the novel context condition, each rat was placed individually in a dark holding room for 30 min (to which they had received two 30 min familiarisation sessions on the preceding days). They were then taken into the novel test room and placed individually inside an activity test cage for 20 min. (Due to an equipment malfunction, two activity scores (one Peri, one Sham) were not recorded.) These rats were then returned to the dark holding room. For the baseline condition, individual rats remained throughout in their home cages without exposure to the dark room prior to perfusion.

### Perfusion and tissue sectioning

For the novel context condition, rats were perfused 90 min after being returned to the dark holding room. This interval is within the time period when the expression of Fos, the protein product of c-*fos*, peaks, that is, between 60 and 120 min after the inducing event (Bisler et al., 2002; Zangenehpour and Chaudhuri, 2002). Animals in the baseline control condition were taken directly from their home cage immediately prior to perfusion. All rats then received a lethal overdose of sodium pentobarbital (60 mg/kg, Euthatal, Marial Animal Health, Harlow, Essex, UK) and were transcardially perfused with 0.1 M PBS followed by 4% paraformaldehyde in 0.1 M PBS (PFA). Brains were removed from the skull, postfixed in PFA for 4 h, and then incubated in 25% sucrose at room temperature overnight on a stirrer plate. The brains were cut in the coronal plane into 40-µm sections using a freezing microtome. A series of one in four sections were collected in PBS and then stained with cresyl violet (a Nissl stain), while another one in four series was retained for immunohistochemistry.

### Lesion analysis

Only one hemisphere in each Peri brain was analysed for Fos expression, while the other was eliminated. This procedure ensured that Fos counts were only taken from those hemispheres with either no evidence of surgically induced hippocampal cell loss or with loss restricted to just one coronal section (typically in the hippocampal subfield, CA1). Brains that suffered damage to both hippocampi were excluded from the study. In those hemispheres analysed for study, the boundaries of the lesions were drawn onto five coronal plates (bregma: −2.80 to −6.72 mm) from Paxinos and Watson (2005). These images were then scanned, and the area of damage was calculated using cellSens Dimension Desktop, version 1.12 (Olympus, Southend-on-Sea, UK).

### Immunohistochemistry

Brain sections were initially stored at −20°C in cryoprotectant. Free-floating sections were then immunohistochemically stained with sections from one rat from each of the four behavioural groups placed in the same reaction vessel, that is, sections from all four groups were processed concurrently. This arrangement sought to decrease staining variation between groups. The sections were first washed six times in PBS to remove the cryoprotectant, then washed in 0.2% Triton-X 100 in 0.1M PBS (PBST), once in 1% H_2_O_2_ in PBST (to block endogenous peroxidases), and then four further times in PBST. The sections were then incubated in a blocking solution of 3% normal goat serum (NGS) in PBST for 1 h followed by the primary antibody solution; rabbit-anti-c-*fos* (1:15,000) and 1% NGS diluted in PBST (Cat# PC38; Calbiochem; now part of Merek Millipore, Nottingham, UK), for 48 h at 4°C. The sections were then washed four times in PBST, before being incubated in the secondary antibody solution; biotinylated goat-anti-rabbit (1:200; Vector Laboratories, Peterborough, UK) diluted in 1.5% NGS in PBST for 2 h at room temperature. The sections were washed four times in PBST. They were then incubated in avidin-biotinylated horseradish peroxidase complex in PBST (Elite kit; Vector Laboratories) for 1 h at room temperature. The sections were washed four times in PBST and then twice in 0.05 M Tris buffer (pH 7.4). All washes were 10 min unless otherwise stated. Finally, diaminobenzidine (DAB substrate kit; Vector Laboratories) was used as the chromogen to visualise the location of immunostaining. The reaction was stopped in cold PBS. The sections were mounted onto double gelatine-subbed glass slides and allowed to air dry for at least 48 h, dehydrated in increasing concentration of alcohol washes, cleared in xylene, and coverslipped using DPX as the mounting media.

### Image capture and analysis of c-fos activation

Images from each region of interest (ROI) were captured from six consecutive sections (each 120 µm apart) from one hemisphere per animal. The equivalent hemisphere (left or right) was also analysed in the corresponding ‘Sham’ control animal. Image capture used a 5× objective lens (numerical aperture of 0.12) on a Leica DMRB microscope with an Olympus DP70 camera. The field of view was 0.84 × 0.63 mm, so that cortical regions only required one image per section to include all lamina. For the hippocampus, multiple images were taken and combined (Microsoft Image Composite Editor (ICE); Microsoft, Redmond, WA, USA). Using ANALYSIS^D software (Soft-Imaging Systems, Olympus Corporation). Fos-positive cells were quantified by counting the number of immunopositive nuclei (mean Feret diameter of 4−20 µm) stained above a greyscale threshold set 60−70 units below the peak grey value measured by a pixel intensity histogram.

### ROIs

**Figure 2.**
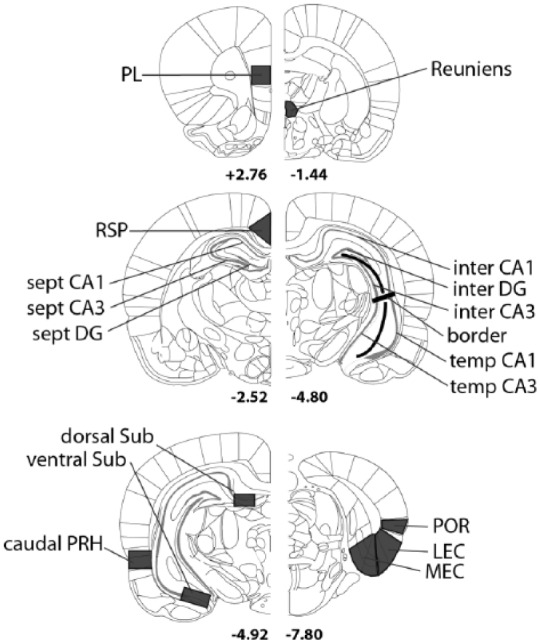
Regions of interest for c-*fos* analyses. Sites examined: CA fields – intermediate (inter), septal (sept), and temporal (temp); dentate gyrus (DG); dorsal subiculum (dorsal Sub); lateral entorhinal cortex (LEC); medial entorhinal cortex (MEC); prelimbic cortex (PL); perirhinal cortex (PRH); postrhinal cortex (POR); nucleus reuniens of thalamus (Reuniens); retrosplenial cortex (RSP); and ventral subiculum (ventral Sub). The numbers below refer to the approximate distance in millimetre from bregma. Source: Adapted from the atlas of Paxinos and Watson (2005).

The borders of the perirhinal and postrhinal cortices follow the description of Burwell and Amaral (1998a; see also Burwell, 2001), while those of the other brain areas correspond to Swanson (1992). The AP coordinates (mm from bregma) given in the descriptions below and in Figure 2 are from Paxinos and Watson (2005). The regional groupings are those subsequently used in the statistical analyses of Fos counts.

#### Hippocampal subfields

Hippocampal subfields (dentate gyrus, CA1, and CA3) were subdivided into their septal (dorsal), intermediate (dorsal), and temporal (ventral) divisions(Bast, 2007; Strange et al., 2014). The septal hippocampus counts (dentate gyrus, CA3, and CA1) were obtained from sections from AP −2.52 to −3.24, while those for the intermediate dorsal hippocampus (dentate gyrus, CA1, and CA3) came from sections between AP −4.80 and −5.52. The border between the dorsal intermediate and temporal hippocampus corresponds to −5.0 mm ventral from bregma (see Figure 2; Paxinos and Watson, 2005). Within the temporal hippocampus, counts were made in the CA1 and CA3 fields at the same AP as the intermediate dorsal hippocampus (note that the dentate gyrus is not present at this level). Additional Fos-positive cell counts were taken in both the dorsal and ventral subiculum (from around AP −5.16).

#### Parahippocampal cortices

Separate Fos-positive cell counts were taken from the LEC and MEC, as well as the postrhinal cortex. The LEC counts were taken from more caudal parts of the area to ensure that there was no encroachment from the perirhinal lesion in the Peri groups. In the Sham cases only, Fos-positive cell counts were made in the caudal perirhinal cortex (areas 35 and 36; see Burwell, 2001). This caudal portion (from AP −4.80 to −5.52) was selected as previous studies indicate that this region is particularly involved in processing novel visual stimuli (Albasser et al., 2009, 2010; Kinnavane et al., 2014; Olarte-Sánchez et al., 2014).

#### Other hippocampal-related areas

Fos-positive cell counts were made within the prelimbic cortex (PL; AP +3.72 to +2.76), the granular retrosplenial cortex (RSP; AP −2.28 to −3.36), and nucleus reuniens (AP −1.44 to −2.28). The granular retrosplenial cortex (area 29) was selected as it is both the principal recipient of the projections from the hippocampal formation to this region and the source of its projections to entorhinal cortex (Van Groen and Wyss, 1990, 1992, 2003). There are also direct projections from the temporal region of CA1 and the subiculum to prelimbic cortex, with return projections via nucleus reuniens of the thalamus (Conde et al., 1995; Prasad and Chudasama, 2013; Vertes et al., 2007).

### Statistical analysis

#### Behavioural results

Activity scores from the ‘novel’ groups were separated between ‘same beam’, that is, a single beam being repeatedly broken, and ‘beam crossovers’, that is, the front and back beams broken sequentially. These data were compared with a one between-subject factor (surgical condition) and one within-subject factor (‘same beam’ or ‘beam crossovers’) analysis of variance (ANOVA). The total number of beam breaks across the 20-min exposure to the novel context was then divided into four bins of 5 min and compared with a one between-subject factor (surgical condition) and one within-subject factor (bin).

#### Fos-positive cells counts

To analyse group differences (Sham vs Peri lesion; baseline vs novel context) in the ROIs, two between-subject factors (surgical condition and baseline/novel context) and one within-subject factor (ROI) ANOVA was calculated. This analysis was carried out separately for three regional groupings: (1) hippocampal subfields, (2) parahippocampal cortices, and (3) other hippocampal-related areas. These regional groupings helped to reduce type 1 errors by limiting the number of comparisons. The Fos counts in perirhinal cortex (Sham groups only) were compared using a one between-subject (baseline/novel context) by one within-subject factor (areas 35 and 36) ANOVA. When an interaction was significant (p ⩽ 0.017 corrected for multiple tests), simple effects were examined.

While the Fos counts from the novel groups were normally distributed, cell counts from both baseline control groups (‘Peri Baseline’ and ‘Sham Baseline’) were not (Shapiro–Wilk test). As the baseline Fos counts in all ROIs were positively skewed and their means were proportional to their variance, a square-root transformation was applied to the data (Howell, 2011) when the analyses involved only these groups. Other analyses involving all four groups used the raw Fos counts for comparability, mindful that ANOVA is relatively robust to violations of the normality assumption when group sample sizes are equal (Howell, 2011).

Pearson product-moment correlation coefficients were calculated for the Fos-positive cell counts in the various sites, as well as with the activity of animals in the novel context condition. In both baseline control groups, the Pearson product-moment correlation coefficients were calculated based on the transformed scores as these data were subsequently used for SEM, where normality is assumed (Arbuckle, 2011).

### Structural equation modelling

SEM uses multiple-equation regression models to quantify potentially causal relationships between sets of variables in a theoretical structure, thereby testing models that can include the potential direction of effects (Schumacker and Lomax, 2010). (In some cases, a direction of effect could not be inferred as the fit of the models did not change when the path direction was reversed. This situation is indicated in the figures by a double-headed arrow.) The SEM software package, SPSS AMOS version 20.0 (IBM Corp, Armonk, NY, USA) computed the analyses. Maximum likelihood estimation, which is recommended for use with smaller sample sizes (Arbuckle, 2011), allowed the programme to estimate effects among variables. All models tested were based on well-established anatomical connections (Furtak et al., 2007; Van Strien et al., 2009; Witter, 2002).

An anatomically plausible model was specified and the covariance matrix of the regional Fos counts estimated the strength of the relationship (path) between regions as set out in this model. The path coefficient of a connection between two regions (Arbuckle, 2011) estimates the ‘effective connectivity’ or the extent to which one region directly influences the other (Protzner and McIntosh, 2006). Models were assessed based on how well the implied (estimated) variance–covariance matrix replicates the sample (observed) variance–covariance matrices of the observed data (Schumacker and Lomax, 2010). A model with good fit has a non-significant χ^2^, and the ratio of χ^2^ to the degrees of freedom is <2 (Tabachnick and Fidell, 2001). The comparative fit index (CFI) and the root mean square error of approximation (RMSEA) are additional measures of fit that are applicable for smaller sample sizes (Fan and Wang, 1998; Hu and Bentler, 1998). A CFI of >0.9 is considered acceptable (Schumacker and Lomax, 2010), while a RMSEA of <0.08 is considered acceptable (Tabachnick and Fidell, 2001). Given the relatively small group sizes, each model should contain twice as many cases as the number of variables to be estimated (Bollen and Long, 1992; Wothke, 1993). Finally, the squared multiple correlation (R^2^ or coefficient of determination) is presented, indicating the amount of variance in each brain region accounted for by the model (Arbuckle, 2011).

The various groups were compared on the same network model by a stacking procedure. In this procedure, the path coefficients of all paths in the model are constrained so that they must have the same value for all groups, creating a ‘structural weights model’. If the model fit when the paths are constrained is significantly worse than when the paths are free to have different values for each group (as determined by a χ^2^ difference test), this indicates that the paths differ among the groups (Protzner and McIntosh, 2006; Schumacker and Lomax, 2010). Subsequently, each path can be independently unconstrained and the fit compared to the structural weights model, again using a χ^2^ difference test to determine in which path the difference occurs (Protzner and McIntosh, 2006). When there were marked differences in the overall fit of the same model between two or more groups, the alpha level of the first χ^2^ difference test was slightly relaxed in order to explore the potential reasons why one group had poor fit.

## Results

The histological and behavioural analyses only relate to those animals used for the c-*fos* analyses. As explained, the findings came from two cohorts of rats. The data from these two cohorts were repeatedly compared, though the outcome is only presented when there was a significant cohort difference (p ⩽ 0.05). Rats from both cohorts populated all conditions.

### Lesion analysis

Based on the exclusion criteria (see section ‘Lesion analysis’), seven animals were removed from group Peri Novel and three were excluded from Peri Baseline. Following these exclusions, the group numbers were as follows: Peri Novel, n = 11 (3 from cohort A, 8 from cohort B); Peri Baseline, n = 12 (6 from cohort A, 6 from cohort B); Sham Novel, n = 11 (4 from cohort A, 7 from cohort B); Sham Baseline, n = 12 (7 from cohort A, 5 from cohort B). Group Peri Novel contained six left and five right hemispheres. For group Peri Baseline, three left and nine right hemispheres were analysed. The corresponding hemispheres were analysed in the matched Sham surgical controls.

**Figure 3.**
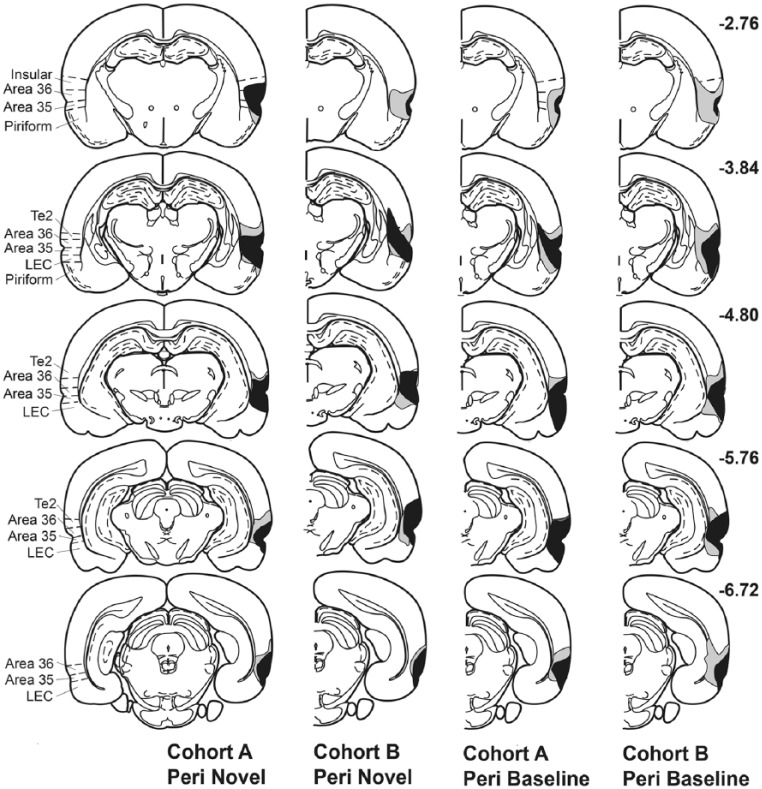
Perirhinal lesion reconstructions. Diagrammatic reconstructions of the perirhinal cortex lesions showing the individual cases with the largest (grey) and smallest (black) lesions for rats from cohorts A and B in groups Peri Novel and Peri Baseline. The left panel illustrates regions involved for comparison. Sites highlighted: areas 35 and 36 of the perirhinal cortex, insular cortex, lateral entorhinal cortex (LEC), and piriform cortex. The numbers refer to the distance (in mm) from bregma (note that the hemispheres analysed came from both right and left hemispheres). Source: Adapted from Paxinos and Watson (2005).

The lesions involved much of the AP extent of perirhinal cortex, with only small regions of tissue sparing (Figure 3). The extent of perirhinal tissue loss in those hemispheres analysed for Fos expression in the Peri Novel context condition ranged from 40.6% to 73.9% (cohort A) and 67.8% to 98.5% (cohort B). The corresponding extent of tissue loss in the Peri Baseline condition ranged from 70.7% to 89.9% (cohort A) and 68.6% to 100% (cohort B). The extent of tissue loss did not differ between the Peri Novel and Peri Baseline groups (t < 1).

The attempt to make near-complete perirhinal cortex lesions led to some extra-perirhinal damage. This additional damage was typically in the most ventral parts of area Te2 and the most dorsal parts of the piriform cortex and LEC, that is, those cortical areas immediately adjacent to perirhinal cortex (Figure 3).

### Behavioural testing

Analyses of the beam breaks over the 20 min session (‘same beam’ and ‘beam crossovers’) found no overall effect of perirhinal cortex lesions (F(1, 18) = 1.36, p = 0.26). Similarly, there was no interaction between lesion and type of beam break (F(1, 18) = 3.21, p = 0.09). When the beam breaks were divided into 5 min bins, the activity levels showed a highly significant reduction across the 20 min session (F(3, 36) = 11.4, p < 0.001), with no effect of surgery (F < 1) and no interaction between these factors (F < 1). Both the perirhinal lesion and sham control rats showed a significant decrease in activity. (Note: these data were only available for 14 rats from cohort B.) This reduction in activity is assumed to principally reflect habituation to a novel environment.

### Comparisons of Fos-positive cell counts

#### Hippocampal subfields

**Figure 4.**
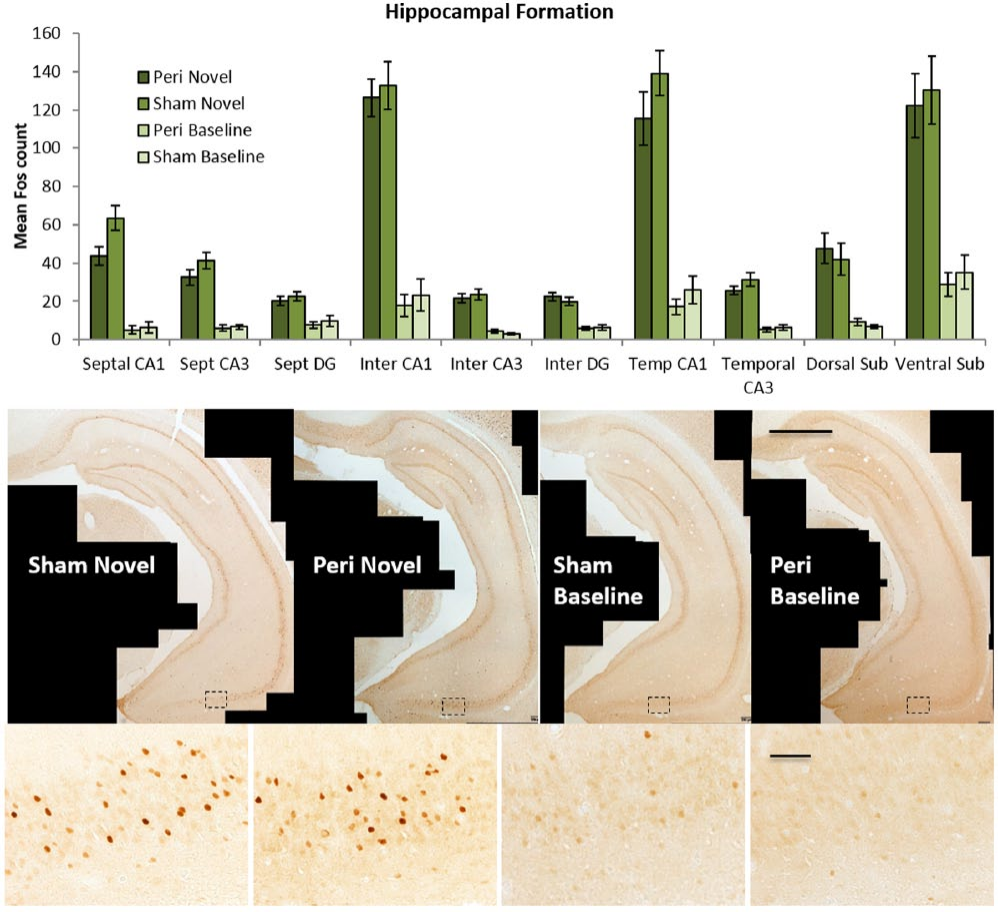
Mean Fos-positive cell counts per group in the hippocampal formation. Top panel: Graph of results from all hippocampal sites analysed: CA fields – intermediate (inter), septal (sept), and temporal (temp); dentate gyrus (DG); and subiculum (Sub). Exposure to a novel context reliably increased Fos-related activity in all regions analysed (p < 0.001). Data are presented as means ± SEM. Middle panel: Representative photomicrographs from coronal sections that depict Fos-positive cells in intermediate and temporal levels of the hippocampus for all behavioural conditions. Scale bar: 1000 µm. Bottom panel: Higher magnification photomicrographs of regions corresponding to the dashed rectangle of the photomicrograph above. Scale bar: 50 µm.

Being placed in the novel context dramatically increased c-*fos* activity, although the perirhinal cortex lesions had no apparent effect on the mean Fos counts in the hippocampal formation (Figure 4). A significant Mauchly test (p ⩽ 0.001) indicated that the assumption of sphericity of the within-subject variable (ROI) was violated and so Greenhouse–Geisser corrected degrees of freedom and p-values are presented for the within-subject comparisons (Howell, 2011).

Hippocampal Fos counts in the novel context rats were consistently considerably higher than those of the rats in the baseline (home-cage) controls (F(1, 42) = 166, p ⩽ 0.001), with individual areas showing different levels of Fos expression (F(2.8, 116) = 101.1, p ⩽ 0.001; Figure 4, upper panel). There was a significant context by subfield interaction (F(2.8, 116) = 48.2, p ⩽ 0.001) as the increase in Fos counts from baseline to novel context seemingly differed among subfields (this novelty difference was highly significant in all subfields, F(1, 42) > 27, p ⩽ 0.001; Figure 4, upper panel). While this increase seemed most evident in CA1, scaling effects were present. Comparisons of Fos-positive cell counts across the 10 hippocampal subfields found no overall effect of perirhinal lesions (F(1, 42) = 1.43, p = 0.24; Figure 4, upper panel). Similarly, there was no lesion by context interaction (F < 1), lesion by subfield interaction (F(2.8, 116) = 1.12, p = 0.34), or three-way interaction (F < 1).

#### Parahippocampal cortices

**Figure 5.**
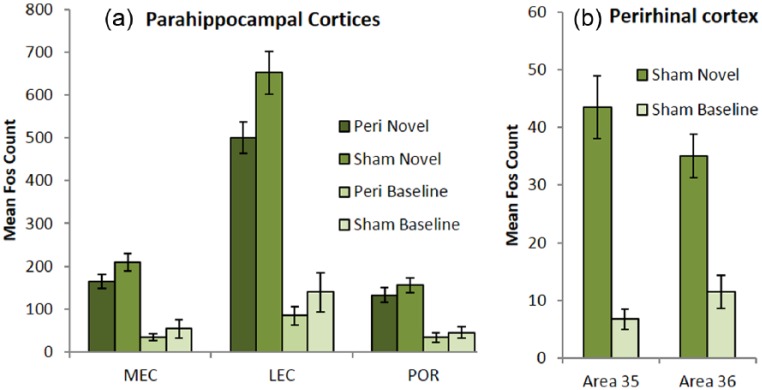
Mean Fos-positive cell counts per group in the parahippocampal formation. (a) Sites analysed in all four groups: lateral entorhinal cortex (LEC), medial entorhinal cortex (MEC), and postrhinal cortex (POR). (b) Sites analysed in only the Sham controls: areas 35 and 36 of the perirhinal cortex. Exposure to a novel context reliably increased Fos-related activity in all regions analysed (p < 0.001). Data are presented as means ± SEM.

Overall, novel context exploration produced higher Fos counts in the MEC, LEC, and postrhinal cortex than remaining in the home cage (F(1, 40) = 113, p ⩽ 0.001; Figure 5(a)). Rats with perirhinal cortex lesions had lower Fos-positive cell counts across the parahippocampal cortices, with the LEC seemingly most affected (Figure 5(a)). As in the hippocampus, the assumption of sphericity was violated, and so Greenhouse–Geisser corrected degrees of freedom and p-values are presented (Howell, 2011).

The context manipulation differentially affected the three cortical areas (F(1.2, 49) = 113.5, p ⩽ 0.001) although simple effects revealed that MEC, LEC, and postrhinal cortex all had higher Fos-positive cell counts when exposed to the novel context compared to baseline (all F > 46.8, p ⩽ 0.001; Figure 5(a)). Numerically, this increase appeared greatest in LEC, although this may have been due to scaling effects generated by the comparatively higher Fos counts in LEC than in MEC or the postrhinal cortex. For perirhinal cortex (Figure 5(b)), counts could only be made in the two Sham groups. Once again, the novel context condition raised Fos counts (F(1, 21) = 41.7, p ⩽ 0.001; Figure 5(b)). Overall, the Fos counts in areas 35 and 36 did not differ (F < 1), although the context manipulation affected the two areas differently (F(1, 21) = 9.84, p = 0.005) with a numerically greater Fos increase in area 35 (F(1, 21) = 44.2, p ⩽ 0.001) than area 36 (F(1, 21) = 25.5, p ⩽ 0.001; Figure 5(b)).

Across the three parahippocampal regions analysed in all four groups, Fos counts were numerically lower in the Peri rats than in the Sham controls (Figure 5(a)). While this contrast did not reach the corrected levels of significance (F(1, 40) = 5.41, p = 0.025), there was a significant region by lesion interaction (F(1.2, 49) = 6.00, p = 0.013). This interaction indicated that the various parahippocampal areas were differentially affected by the perirhinal lesions. Simple effects revealed that this interaction reflected decreased Fos counts in the LEC of the rats with perirhinal lesions (F(1, 40) = 6.56, p = 0.014; Figure 5(a)), a lesion effect that did not extend to the MEC or postrhinal cortex (F(1, 40) = 3.44, p = 0.071; F(1, 40) = 1.59, p = 0.21, respectively). Finally, the three-way interaction (area, lesion, and context) was non-significant (F(2, 80) = 1.61, p = 0.21).

#### Other hippocampal-related areas

**Figure 6.**
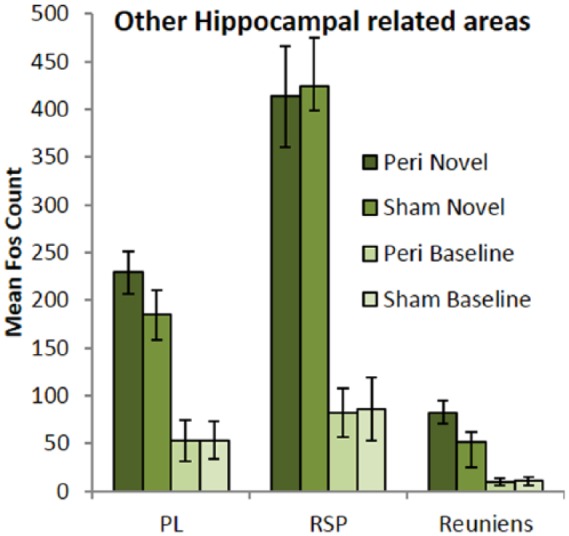
Mean Fos-positive cell counts per group in other hippocampal-related areas. Sites analysed: prelimbic cortex (PL), retrosplenial cortex (RSP), and nucleus reuniens of the thalamus (Reuniens). Exposure to a novel context reliably increased Fos-related activity in all regions analysed (p < 0.001). Data are presented as mean ± SEM.

While exposure to a novel context dramatically increased Fos expression (F(1, 42) = 74.7, p ⩽ 0.001) in all three areas (Figure 6), the perirhinal cortex lesions did not affect overall Fos activity in the prelimbic cortex, retrosplenial cortex, or nucleus reuniens of the thalamus (F < 1). While Fos counts differed between areas (F(2, 84) = 105.9, p ⩽ 0.001), there was no lesion by context interaction (F < 1) or region by lesion interaction (F < 1). Although the context by region interaction was significant (F(2, 84) = 49.2, p ⩽ 0.001), with the retrosplenial cortex showing a greater increase than prelimbic cortex, the relatively low counts in nucleus reuniens created a scaling effect.

#### Activity behaviour and Fos-positive cell counts

For each of the two relevant groups (Sham Novel and Peri Novel), only one site had an initial significant correlation (p < 0.05). In both cases, the Fos-positive cell counts correlated positively with ‘same beam’ breaks (Sham Novel, intermediate CA3, r = 0.75, p = 0.012: Peri Novel, temporal CA3, r = 0.63, p = 0.038). However, in neither case did these effects survive correction for multiple comparisons, suggesting that the Fos counts were not a direct product of the amount of locomotor activity.

### Structural equation modelling

Initial inspection of all of the inter-area correlations revealed an apparent difference between the novel context and baseline control conditions. For both the Sham Baseline group (120/170) and the Peri Baseline group (87/120), a large majority of the inter-area Fos count correlations were significant at an uncorrected level (~70% at p < 0.05). In contrast, for both the Sham Novel group (41/170) and the Peri Novel group (32/120), the corresponding proportion was much lower (~25%, i.e. almost one-third of the number).

For SEM, all of the networks examined had to have anatomical plausibility with respect to their interconnections and the direction of these connections. A valid model of context learning would be expected to have good fit for the novel context condition but not the baseline (home-cage) condition.

*1. Is novel context exposure associated with specific network patterns of c-fos activity predicted by the BIC framework and is this affected by perirhinal cortex damage?* The first model to be tested used the parahippocampal (postrhinal)–medial entorhinal network described by Ritchey et al. (2015). In this refined version of the BIC framework, interactions between the parahippocampal (postrhinal) cortices and retrosplenial cortex are included, creating what is referred to as the posterior–medial (PM) system (Figure 1). For this initial analysis, the Fos counts along the longitudinal hippocampal axis were combined, that is, the temporal, intermediate, and septal subregions of the dentate gyrus, CA3, and CA1. This decision reflects the way in which a coronal section across entorhinal cortex will include connections along the full longitudinal axis of the hippocampus (Furtak et al., 2007; Van Strien et al., 2009). A maximum of six nodes could be included in each model given the sample size (Bollen and Long, 1992; Wothke, 1993).

**Figure 7.**
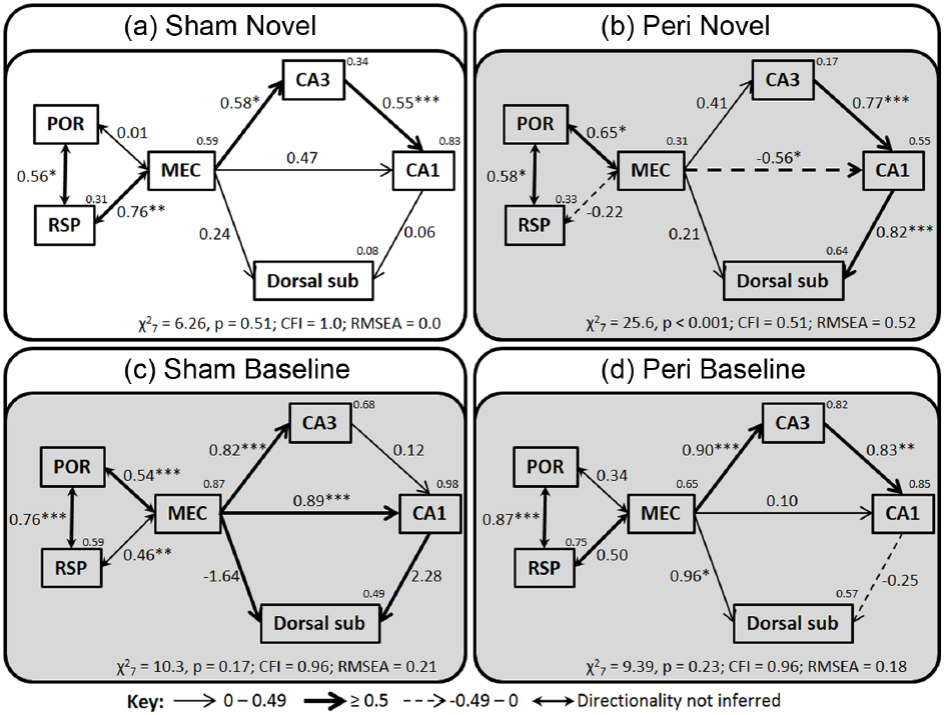
Testing the posterior–medial system of the BIC framework. (a) The posterior–medial system has good fit for group Sham Novel. (b) The same network model for group Peri Novel has poor fit. The same model also has poor fit for (c) group Sham Baseline and (d) group Peri Baseline. Model fit is noted at the bottom of each model (comparative fit index (CFI); root mean square error of approximation (RMSEA)). The strength of the causal influence of each path is denoted both by the thickness of the arrow and by the path coefficient next to that path. The number above the top right corner of each area box is the R^2^ value, denoting the variance accounted for by the inputs to that region. Sites depicted: medial entorhinal cortex (MEC); postrhinal cortex (POR); retrosplenial cortex (RSP); and hippocampal subfields CA1, CA3, and dorsal subiculum (sub). (Models with a grey background have poor fit.) *p < 0.05; **p < 0.01; ***p < 0.001.

This PM system, which is depicted in Figure 7, was found to have good fit for group Sham Novel context ($\chi_{7}^{2}=6.26$, p = 0.51; CFI = 1.0; RMSEA = 0.0; Figure 7(a)). In contrast, the same model did not fit the Sham Baseline Fos data ($\chi_{7}^{2}=10.3$, p = 0.17; CFI = 0.96; RMSEA = 0.21; Figure 7(c)). When compared directly by stacking the data from these two groups on the same model, the model in which the path coefficients were all free to vary did not have significantly better fit than the model in which all path coefficients were constrained to be the same for both groups ($\chi_{8\mathrm{Diff}}^{2}=13.87$, p = 0.085). While this indicates that the Fos activity data of the two groups did not differ between the regions set out in the model, this contrast was close to the level of significance, and as the model had poor fit for group Sham Baseline, further examination took place. When the pathways that compose the model were individually unconstrained, the functional connection between postrhinal cortex and MEC was found to be stronger in the Sham baseline group ($\chi_{1\mathrm{Diff}}^{2}=4.92$, p = 0.027), while the functional connection between CA3 and CA1 was stronger in group Sham Novel ($\chi_{1\mathrm{Diff}}^{2}=6.67$, p = 0.009; all other paths: $\chi_{1\mathrm{Diff}}^{2}<1.9$).

The specificity of the PM system was tested in two further ways. First, we tested the complementary item division of the updated BIC framework, that is, the anterior–temporal (AT) system (Figure 1; see Ritchey et al., 2015). Fos counts from the perirhinal entorhinal cortex and LEC replaced those from the postrhinal entorhinal cortex and MEC, while the ventral subiculum replaced the dorsal subiculum. This AT model had only poor fit for group Sham Novel ($\chi_{4}^{2}=6.74$, p = 0.15; CFI = 0.71; RMSEA = 0.26).

Second, data were taken from a previous c-*fos* experiment that matched this study in all respects, except for one critical feature. Rats in that experiment were exposed to multiple novel object recognition problems in a familiar environment (Kinnavane et al., 2016). Consequently, activity should be biased towards the AT item system, that is, the perirhinal cortex and LEC. Consistent with that prediction, models based on the AT system had good fit (Kinnavane et al., 2016). However, when the postrhinal–medial entorhinal network was tested using the Fos counts from that same object recognition study (Kinnavane et al., 2016), the resulting model was of very poor fit ($\chi_{4}^{2}=15.2$, p = 0.004; CFI = 0.74; RMSEA = 0.50). Surprisingly, when the same postrhinal–medial entorhinal network was applied to the control condition from that study, which involved novel objects but no familiarity discrimination, the model retained its fit ($\chi_{4}^{2}=3.23$, p = 0.52; CFI = 1.0; RMSEA = 0.0).

Finally, evidence that the perirhinal lesions disrupted the PM system of the BIC framework (Figure 7) came from the finding that the Fos data from the Peri Novel group had only poor fit ($\chi_{7}^{2}=25.75$, p = 0.001; CFI = 0.51; RMSEA = 0.52). Consistent with the above results, the updated BIC framework also failed to fit the data from group Peri Baseline ($\chi_{7}^{2}=9.39$, p = 0.27; CFI = 0.96; RMSEA = 0.18).

**Figure 8.**
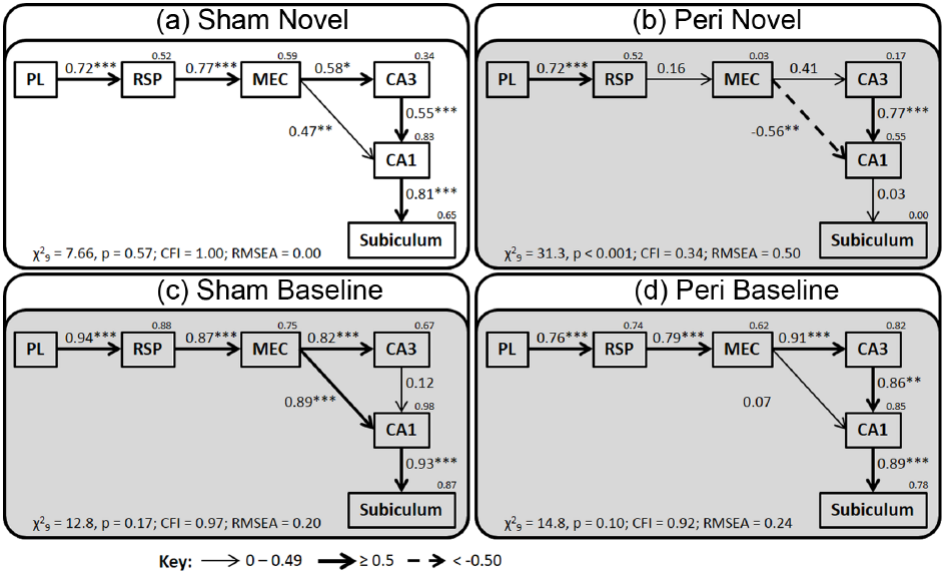
Optimal model for group Sham Novel. (a) This network, which is expanded to include prelimbic cortex, has optimal fit for data from group Sham Novel. (b) The same network for group Peri Novel has poor fit. The same model also has poor fit for group (c) Sham Baseline and (d) group Peri Baseline. Model fit is noted at the bottom of each model (comparative fit index (CFI); root mean square error of approximation (RMSEA)). The strength of the causal influence of each path is denoted both by the thickness of the arrow and by the path coefficient next to that path. The number above the top right corner of each area box is the R^2^ value, denoting the variance accounted for by the inputs to that region. Sites depicted: medial entorhinal cortex (MEC); prelimbic cortex (PL); retrosplenial cortex (RSP); and hippocampal subfields CA1, CA3, and dorsal subiculum (sub). (Models with a grey background have poor fit.) *p < 0.05; **p < 0.01; ***p < 0.001.

*2. What is the optimal model for the Sham Novel context group?* For group Sham Novel Context, the optimal model involved many of the regions implicated in the PM network of the updated BIC framework ($\chi_{9}^{2}=7.66$, p = 0.57; CFI = 1.00; RMSEA = 0.00; Figure 8(a)). Interestingly, the prelimbic and retrosplenial cortices had better predictive value when positioned early in the model. All of the paths in the model were significant, and all paths have directionality as the fit of the model was worse when the direction of each path was reversed. It should be noted that the hippocampal Fos data presented here are counts combined along the longitudinal hippocampal axis. If dorsal CA3 and CA1 counts are substituted for the combined counts, the model retains acceptable but inferior fit. Whereas if ventral (temporal) CA3 and CA1 counts are substituted, this produces a poorly fitting model (data not presented).

This same optimal network model did not have acceptable levels of fit for any of the other three behavioural groups (Figure 8(b)–(d)). When the two intact groups were directly compared by stacking their Fos data on the same model (Figure 8(a)), the overall group difference between model fit was close to being significant ($\chi_{6\mathrm{Diff}}^{2}=11.5$, p = 0.075). As the model had poor fit for group Sham Baseline, additional analyses were conducted. When each of the component paths was allowed to vary individually, only freeing the paths from retrosplenial cortex to MEC ($\chi_{1\mathrm{Diff}}^{2}=4.41$, p = 0.036) and from CA3 to CA1 ($\chi_{1\mathrm{Diff}}^{2}=6.62$, p = 0.010) significantly improved fit (all other paths: $\chi_{1\mathrm{Diff}}^{2}<1.2$). This difference potentially reflects the strengthening of intrinsic hippocampal connections with novel context exploration (Figure 8(a)).

None of the network models with acceptable fit for group Sham Novel transferred over to the Sham Baseline group (e.g. Figures 7 and 8). This failure again suggests that the context-driven models are specific and not simply driven by correlations associated with baseline Fos expression.

Finally, evidence that perirhinal lesions disrupted network activity in the medial temporal lobe came from the fact that it was not possible to generate a network model of acceptable fit with data from group Peri Novel. Additionally, when the data from the two novel context groups (Sham Novel and Peri Novel) were stacked on the optimal model for Sham Novel (Figure 8), the activity-related Fos data differed significantly between the two groups ($\chi_{6\mathrm{Diff}}^{2}=17.6$, p = 0.007). To investigate further, each of the pathways was individually unconstrained revealing significant differences in the steps from MEC to CA1 ($\chi_{1\mathrm{Diff}}^{2}=8.30$, p = 0.004) and CA1 to subiculum ($\chi_{1\mathrm{Diff}}^{2}=4.00$, p = 0.047; Figure 8). Additionally, for group Sham Novel, the correlation between Fos counts in MEC and CA1 was strong and positive (r = 0.79, p = 0.004), whereas in group Peri Novel, this correlation was negative and non-significant (r = −0.24, p = 0.48). Formal comparison of these correlations using Fisher’s r-to-z transformation revealed that these correlations were significantly different (z = 2.6, p = 0.009). Taken together, these results indicate that the perirhinal cortex lesions altered coordinated activity between the entorhinal cortex and CA1 when animals explored a novel context.

## Discussion

This study sought to test networks of interlinked c-*fos* activity associated with context learning in both intact rats and rats with perirhinal cortex lesions. In one condition, rats were placed in a novel environment (unfamiliar cages in an unfamiliar room), and in the other, they remained in their home cages. Although this comparison brings additional changes in locomotor and arousal levels between the two conditions, it has the benefit of creating robust, marked differences in c-*fos* expression, so more reliably testing any impact of perirhinal cortex loss. A further point is that the study did not include additional tests to confirm learning about the novel context, aside from the evidence of habitation that came from the locomotor scores. It should, however, be remembered that context learning is regarded as spontaneous (Dix and Aggleton, 1999; Good et al., 2007) and that the context shift used in this study would be considered highly salient. Consequently, it cannot be excluded that changes in c-*fos* expression may have been driven by those differences in arousal, locomotor activity or anxiety, associated with experiencing a novel context.

The neural networks tested were based on recent refinements of the BIC framework (Diana et al., 2007; Ranganath, 2010; Ritchey et al., 2015) which emphasises relationships between parahippocampal (postrhinal), medial entorhinal, and hippocampal areas for context learning. The resulting PM system (Ritchey et al., 2015) was tested using SEM. Networks closely based on the PM system had good fit for the intact novel context group (Figure 7). Furthermore, the optimal network model for this novel context group incorporated much of the PM system, while also adding further inputs from prelimbic cortex (Figure 8). This optimal novel context model retained its fit when the Fos counts came from just the dorsal hippocampus but not the ventral hippocampus. This result is consistent with the outcome of context reactivation studies based on c-*fos* expression in the dorsal hippocampus (Liu et al., 2012, 2014; Ramirez et al., 2013). The same spatial networks, that is, those based on the PM system (Ritchey et al., 2015), did not have acceptable fit for either of the baseline (home-cage) groups. These null results point to the specificity of the BIC framework for contextual learning.

This specificity was tested in two further ways. First, comparable models were examined using perirhinal cortex and LEC, instead of the postrhinal entorhinal cortex and MEC. A decision was made not to divide the subiculum and CA1 Fos counts based on their distal and proximal locations, in order to ensure that all aspects of the models to be compared were held the same, aside from the introduction of the perirhinal entorhinal cortex and LEC. The resulting analyses, which tested the AT item system of the BIC framework (Ritchey et al., 2015), failed to provide models of acceptable fit in the novel context groups. Second, data were taken from a previous experiment that examined medial temporal c-*fos* activity after a test of object recognition memory, again in rats with perirhinal lesions and their surgical sham controls (Kinnavane et al., 2016). The Fos counts from that surgical sham group (analysis not presented) failed to fit the PM system, but did fit the anterior–temporal system. Somewhat surprisingly, the Fos data from their control group (Kinnavane et al., 2016), which was exposed to novel objects but did not make recognition discriminations, could fit the PM system.

Other evidence for the specificity of the context network models came from the baseline home-cage control groups. A striking feature in both the Sham Baseline and Peri Baseline groups was the high level of correlations between Fos levels in the different areas sampled (around 70% of all sites examined), which contrasted with that found in the novel context groups (both around 25%). In the resting condition, the default state appears to involve widespread levels of inter-correlated activity. This pattern changes in the face of a particular learning challenge, for example, new contextual information. Now, more specific networks become engaged, so decreasing overall site-to-site interactions across multiple brain areas.

This study also assessed the impact of perirhinal cortex lesions on medial temporal lobe c-*fos* activity. Perirhinal lesions did not disrupt the size of the hippocampal Fos increase when rats are moved to a novel context. Similarly, overall levels of c-*fos* expression in prelimbic cortex, retrosplenial cortex, and nucleus reuniens of the thalamus all appeared unaffected by the perirhinal cortex lesions. Perirhinal lesions did, however, reduce c-*fos* expression in the parahippocampal region, an effect most apparent in the LEC. This result closely matches the findings from a related study that used object recognition to examine the impact of perirhinal lesions on c-*fos* expression (Kinnavane et al., 2016). The common finding of LEC hypoactivity underlines the particularly close anatomical and functional links between the perirhinal cortex and this entorhinal division (see also Burwell and Amaral, 1998a; Wilson et al., 2013a, 2013b; Witter et al., 2000). Further evidence of perirhinal lesion effects came from the repeated failure to find medial temporal networks of acceptable fit for the Peri Novel group.

Kent and Brown (2012) suggest that the perirhinal cortex role in item processing extends to learning about complex features within contextual surroundings based on unitising stimulus representations. Support comes from evidence that perirhinal lesions can impair fear conditioning to complex auditory cues, as well as contextual conditioning (Bucci et al., 2000, 2002; Burwell et al., 2004; Corodimas and LeDoux, 1995; Kholodar-Smith et al., 2008a, 2008b; Lindquist et al., 2004; Sacchetti et al., 1999). In contrast, perirhinal lesions spare fear conditioning to continuous tones (Bucci et al., 2000; Kholodar-Smith et al., 2008a; Lindquist et al., 2004). Additionally, increased c-*fos* expression in the perirhinal cortex is associated with context shifts (VanElzakker et al., 2008; Vann et al., 2000), as well as with contextual fear conditioning, but not cued fear conditioning (Albrechet-Souza et al., 2011). Thus, the perirhinal cortex may be involved in discriminating and, hence, helping to bring together novel components within a given context, even though this cortical area may be insensitive to their relative spatial disposition (Aggleton et al., 2010; Deshmukh et al., 2012; Jenkins et al., 2004; Wan et al., 1999). It is presumably this latter aspect, along with the relative preservation of inter-hippocampal activity, as seen in this study (see also Kinnavane et al., 2016), which helps to explain why perirhinal cortex lesions often spare those tests of allocentric spatial memory that are highly sensitive to hippocampal damage (Glenn and Mumby, 1998; Machin et al., 2002; Ramos, 2013; Winters et al., 2004). Many of these same tests make additional demands on navigation, an ability closely linked with medial entorhinal–hippocampal function, rather than perirhinal cortex (Buzsáki and Moser, 2013).

It would be wrong, however, to infer that perirhinal cortex lesions are without effect on hippocampal spatial processing. In this study, perirhinal lesions altered entorhinal cortex activity, so disrupting parahippocampal–hippocampal interactions, for example, those with CA1. With this result in mind, it may be relevant that electrophysiological studies have shown that while perirhinal lesions do not appear to affect the initial formation of CA1 place fields, their stability is reduced (Muir and Bilkey, 2001). Other reported hippocampal changes following perirhinal lesions are a reduction in the proportion of theta cells, a phase shift in CA1 place cells, and an altered modulation by movement velocity on place cells (Muir and Bilkey, 2003). At the same time, subtle deficits after perirhinal cortex lesions have been reported by some when using spatial tasks sensitive to hippocampal damage (Aggleton et al., 2004; Liu and Bilkey, 1998a, 1998b, 2001; Wiig and Bilkey, 1994a, 1994b). The current data suggest that these milder deficits stem from parahippocampal dysfunctions, such as those that were evident in LEC.

This study set out to test medial temporal models that describe the processing of context information. Expression of c-*fos* revealed inter-related activity networks that closely match those predicted by refinements of the BIC framework. Arguably, one unexpected finding was the direction of influence in the optimal model for the intact novel context group (Figure 8). While all connections were significantly connected, the best fit was obtained when the model progressed from prelimbic cortex to retrosplenial cortex and then to the medial temporal lobe. In practice, the PM contextual system (Ritchey et al., 2015; Figure 1) can accommodate these findings as it includes reciprocal connections between the retrosplenial cortex and medial temporal sites.

A final point concerns the impression that there are distinct parahippocampal pathways for ‘what’ and ‘where’ information. It has been argued that a more nuanced division might be more appropriate, such as that between local and global reference frames (Knierim et al., 2006, 2014). As part of this process, a more complete model will need to include crosstalk between the two putative pathways, reflecting their interconnectivity (Burwell and Amaral, 1998b). Indeed, evidence of lateral entorhinal–medial entorhinal interactions was found in this study. For example, when the Sham Novel context model (Figure 7) is modified so that LEC replaces the postrhinal cortex, the model still retains good fit (not presented). It was also the case that the perirhinal lesions disrupted the PM system. Similarly, there is evidence from single unit recordings that the rat LEC also plays a role in spatial processing, often in relation to item location (Deshmukh et al., 2012; Hunsaker and Kesner, 2013; Knierim et al., 2014; Neunuebel et al., 2013). The conclusion is that while there is a major division of information processing pathways in the medial temporal lobe and beyond, there remain important interactions between these same pathways at multiple levels, including those between parahippocampal areas.
